# *BMC Psychology* reviewer acknowledgement 2014

**DOI:** 10.1186/s40359-015-0060-9

**Published:** 2015-03-03

**Authors:** Catherine M Rice, Diana M Marshall

**Affiliations:** BioMed Central, Floor 6, 236 Gray’s Inn Road, London, WC1X 8HB UK

## Abstract

The editors of BMC Psychology would like to thank all our reviewers who have contributed to the journal in Volume 2 (2014).

**Azimatun Noor Aizuddin**

Malaysia

**Katie Alcock**

UK

**Anna-Karin Andershed**

Sweden

**Glynne Andrew**

UK

**Janelle Beadle**

USA

**Patrizia Bisiacchi**

Italy

**Michael Karl Boettger**

Germany

**Samantha Bouwmeester**

Netherlands

**Philip Boyce**

Australia

**Doug Brock**

USA

**Christian Bröer**

Netherlands

**Brendan Bunting**

UK

**Noriko Cable**

UK

**Emanuele Antonio Maria Cappella**

Italy

**Shana Carpenter**

USA

**Thep Chalermchai**

Thailand

**John Chaplin**

Sweden

**Jimmy Choi**

USA

**Mary Chong**

Singapore

**Kaj Sparle Christensen**

Denmark

**Deirdre Conroy**

USA

**Helen Correia**

Australia

**Janna Cousijn**

Netherlands

**Georgina Cox**

Australia

**James Coyne**

USA

**Cathy Creswell**

UK

**Simone Croft**

UK

**Keith Donohue**

USA

**Pete Ellis**

New Zealand

**Monica Eriksson**

Sweden

**George Georgiou**

UK

**Rebecca Giallo**

Australia

**George Giannakopoulos**

Greece

**Kenneth Gilhooly**

UK

**Donald Goff**

USA

**Thalia Goldstein**

USA

**Michael Gradisar**

Australia

**Gaia Gragnano**

Italy

**Roser Granero**

Spain

**Daniel Gruehn**

USA

**Gareth Hagger-Johnson**

UK

**Phillipa Hay**

Australia

**Craig Hedge**

UK

**Masahiro Heima**

USA

**Sigurd William Hystad**

Norway

**Tihomir Ilic**

Serbia

**Gary Jones**

UK

**Göran Jutengren**

Sweden

**Jenny Kelly**

Australia

**Margaret Kern**

USA

**Dennis Klass**

USA

**Judith Knausenberger**

Germany

**Tina Kretschmer**

Netherlands

**Anne Katrin Külz**

Germany

**Joanna C. Lee**

USA

**Rana Limbo**

USA

**Wolfgang Linden**

Canada

**Andrew Littlefield**

USA

**Arnold Lohaus**

Germany

**Matthew Macfarlane**

Australia

**Chantal Martin-Soelch**

Switzerland

**Barbara Maughan**

UK

**Stella Mavroveli**

UK

**Adrian Meule**

Germany

**Michela Milioni**

Italy

**Robert Miller**

Germany

**Øyfrid Larsen Moen**

Norway

**Ho Sik Moon**

Korea, South

**Erin Morgan**

USA

**Naser Morina**

Switzerland

**Julia Müller**

Switzerland

**Marcus Munafo**

UK

**Urs Nater**

Germany

**Chunping Ni**

China

**Sam Norton**

UK

**Jason Oliver**

USA

**Femi Oyebode**

UK

**Massimiliano Pastore**

Italy

**Stephanie Penney**

Canada

**Laura Petitta**

Italy

**Monique Pfaltz**

Switzerland

**Vitoria Piai**

USA

**Kevin Pile**

Australia

**Belinda Platt**

Germany

**Olga Pollatos**

Germany

**Stephanie Potochnick**

USA

**Livia Puljak**

Croatia

**Michael Pullmann**

USA

**Nalini Ranjit**

USA

**Julia Rao**

USA

**Andrew Rasmussen**

USA

**Rinie Rinie Geenen**

Netherlands

**Stuart Ritchie**

UK

**Reuben Robbins**

USA

**Gabriela Roman**

UK

**Gennaro Ruggiero**

Italy

**Amanda Sacker**

UK

**Samaneh Sadeghi**

UK

**Yasuhisa Sakurai**

Japan

**Cristina Scarpazza**

UK

**Virginia Schmied**

Australia

**André Schulz**

Luxembourg

**Beate M. Schulze**

Switzerland

**Stefania Sette**

Italy

**Kimberley Smith**

Canada

**Fruzsina Soltesz**

UK

**Tobias Stalder**

Germany

**Piers Steel**

Canada

**Lisanne Stone**

Netherlands

**Alexander Strobel**

Germany

**Serge Sultan**

Canada

**Lois Surgenor**

New Zealand

**Phern-Chern Tor**

Singapore

**Yannis Tountas**

Greece

**David Trafimow**

USA

**Gareth John Treharne**

New Zealand

**Michael Ullman**

USA

**Peter H Van Ness**

USA

**Eleonora Volpato**

Italy

**Jelte Wicherts**

Netherlands

**Stefan Wiens**

Sweden

**Gill Windle**

UK

**Tim Windsor**

Australia

**Sharlene Wolchik**

USA

**Stefano Zago**

Italy

